# Population-based Characterization of PTEN Hamartoma Tumor Syndrome

**DOI:** 10.21203/rs.3.rs-8279719/v1

**Published:** 2026-01-29

**Authors:** Ying Ni, Gideon Idumah, Chloe Bautista, Lin Li, Lamis Yehia

**Affiliations:** Cleveland Clinic

## Abstract

PTEN hamartoma tumor syndrome (PHTS) is a cancer predisposition disorder caused by germline *PTEN* variants, yet its full clinical spectrum remains poorly defined due to reliance on highly selected cohorts. Accordingly, PHTS is underrecognized and its prevalence underestimated. Leveraging genomic and electronic health record data from 414,830 participants in the All of Us (AoU) Research Program, we identified 55 individuals with pathogenic or likely pathogenic *PTEN* variants, the majority of whom lacked a prior PHTS diagnosis, underscoring underrecognition in the general population. PHTS affects ~ 1/7500 individuals in this US cohort, which is about 26-folds higher than historical estimates for *PTEN*-related disorder. Compared with carriers of other cancer-related gene variants and noncarriers, *PTEN* variant carriers exhibited the highest cancer prevalence and significantly younger ages at first cancer diagnosis. Phenotype enrichment revealed expected overgrowth-related features as well as previously unreported associations, including adenotonsillar hypertrophy, sleep apnea, acanthosis nigricans, and extreme obesity, suggesting broader systemic involvement than classically appreciated. Variant spectra were consistent across the population-based and clinically-ascertained PHTS cohorts. These findings demonstrate that PHTS is more prevalent, more heterogeneous, and more often undiagnosed than current clinical practice reflects, emphasizing the value of population-scale genomics for comprehensive characterization and earlier detection of PHTS.

## Introduction

PTEN hamartoma tumor syndrome (OMIM 158350) is a cancer predisposition disorder caused by germline variants in the tumor suppressor gene *PTEN* (OMIM 601728).^1, 2^ PHTS encompasses several genetically related conditions, including Cowden syndrome (CS), Bannayan-Riley-Ruvalcaba syndrome (BRRS), and other *PTEN*-related disorders.^1, 2^ Germline *PTEN* variants are associated with increased lifetime risks of breast, thyroid, endometrial, kidney, and colon cancers, as well as melanoma.^3, 4, 5, 6, 7^ Individuals with PHTS also have notably elevated lifetime risks of second primary malignant neoplasms (SMN) compared with the general population.^8, 9^ Intriguingly, although considered a classical tumor suppressor gene, germline pathogenic *PTEN* variants are considered one of the most common genetic causes of monogenic autism spectrum disorder (ASD).^10, 11, 12^ As such, there is a well-established bi-modal distribution of PHTS phenotypes, whereby childhood diagnoses are enriched for ASD and/or other neurodevelopmental disorders (NDD), whereas adult diagnoses are enriched with cancer and overgrowth related phenotypes. Early identification and characterization of the natural history of PHTS are important, as prompt recognition facilitates gene-informed medical management, including proactive high-risk cancer surveillance and addressing the associated neurodevelopmental features.

Estimating the prevalence of PHTS has historically been challenging, mainly due to the wide phenotypic and genotypic variability, as well as the association with clinical features that are relatively common in the general population. Hence, many individuals remain undiagnosed, resulting in underestimated prevalence.^2^ A recent study evaluated the prevalence of PHTS in two large research cohorts, suggesting that PHTS may be 10–20 times more common than earlier estimates.^13, 14^ Relatedly, studies characterizing *PTEN* genotype and PHTS-related phenotypes have focused on subsets of individuals from specialized centers, such as our PTEN Multidisciplinary Clinic and Center of Excellence at the Cleveland Clinic, or centers focused on cancer care. Therefore, there are many individuals in the population who remain undiagnosed, and we posit that these individuals may have a different phenotypic spectrum than individuals presenting to specialized clinics due to a diagnosis with or a family history of cancer and/or NDD.

In this study, we leveraged the All of Us (AoU) research program to characterize phenotypes and genotypes of an unselected series of adults with *PTEN*-related syndromes. Additionally, we compared the phenotypic spectrum to that from individuals with pathogenic and/or likely pathogenic (P/LP) variants in other cancer predisposition genes from AoU, and to individuals from the Cleveland Clinic PTEN Multidisciplinary Clinic. Subsequently, we compared the *PTEN* genotype spectrum between AoU participants and those from the Cleveland Clinic PTEN Multidisciplinary Clinic. This effort led to the characterization of *PTEN*-related disorders in a population setting, thus optimizing the identification of PHTS, particularly in individuals unaffected by cancer and/or NDD.

## Results

### Identification of PTEN-related Disorders in All of Us Participants

**Figure 1.**
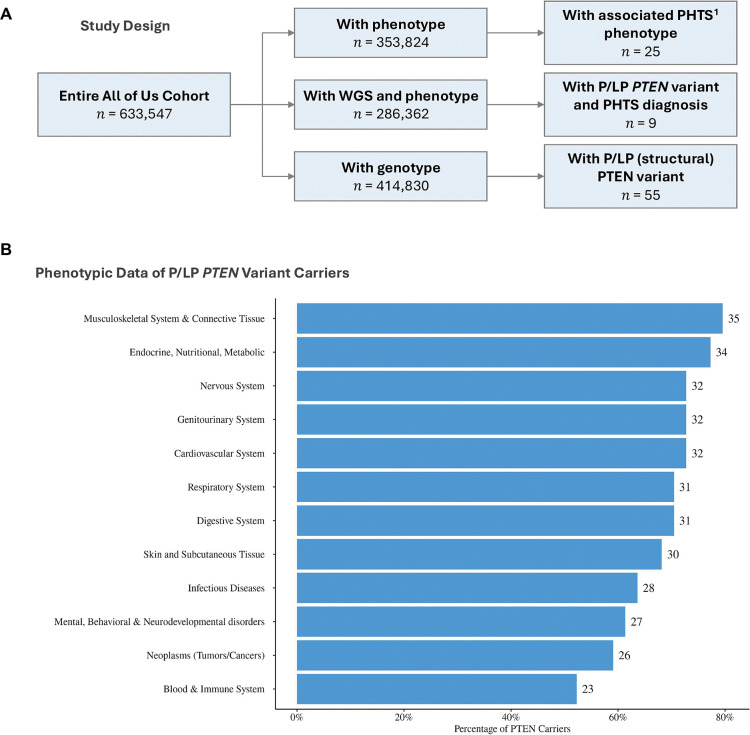
Study Design and Phenotypic Characteristics of Germline *PTEN* Variant Carriers Panel A, ^1^PHTS phenotype include: *PTEN* hamartoma tumor syndrome, Cowden syndrome, Lhermitte-Duclos disease, Bannayan syndrome, Proteus syndrome and Proteus like syndrome. Panel B, phenotypes have been grouped by 22 ICD-10 code chapters, each representing a broad category of conditions and diseases.

For the purposes of phenotype-genotype characterization, we identified a total of 286,362 participants with both srWGS and phenotype data (Fig. 1A). Of the 353,834 participants with phenotype data, 25 individuals were reported to have PHTS, with only a subset reported to have germline P/LP *PTEN* variants. Relatedly, of 414,830 participants with srWGS, 55 individuals were reported to harbor germline P/LP *PTEN* variants (including two individuals with germline structural variants). Of the 46 individuals with EHR data, 37 lacked a formal PHTS diagnosis, suggesting that a subset of individuals with germline P/LP *PTEN* variants remain undiagnosed. A representation of a broad category of conditions grouped by 22 ICD-10 code chapters reveals an overrepresentation of phenotypes associated with the musculoskeletal system and connective tissues, in addition to endocrine, nutritional, and metabolic phenotypes (Fig. 1B). Notably, we did not identify any participant with reported neurodevelopmental disorders, due to limited information about these phenotypes in the AoU dataset.

### Comparison to Participants with Germline Variants in Other Cancer-related Genes

**Figure 2.**
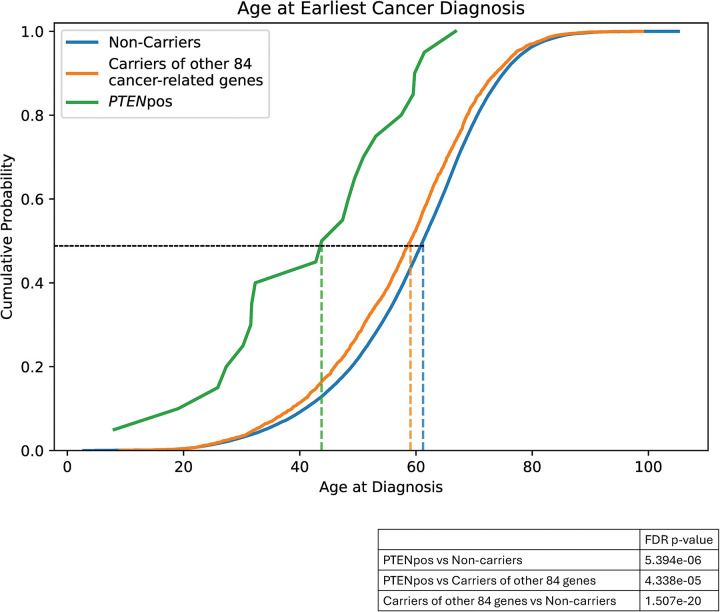
Age at the Earliest Cancer Diagnosis X-axis represents age at diagnosis in years. Non-carrier, participants without germline P/LP variants in the known cancer predisposition genes, including *PTEN*; Other 84 genes, participants with germline P/LP variants in cancer predisposition genes other than *PTEN*; PTEN, participants with germline P/LP *PTEN* variants, including the two participants with structural variants.

In a recent study, we analyzed the AoU database to identify the prevalence of germline P/LP variants in 85 cancer susceptibility genes.^15^ We identified 3,454 unique germline P/LP variants across 77 transcripts and 72 genes, including *PTEN*. Our analysis revealed that 20,968 participants had germline P/LP variants, of which 55 were in individuals with P/LP variants in *PTEN*. Importantly, no participant with germline P/LP *PTEN* variant harbored other germline P/LP cancer susceptibility gene variants. To better characterize the phenotypic spectrum, particularly as related to cancer in a predominantly adult population, we then generated three groups of participants, including those with germline P/LP *PTEN* variants, those with germline P/LP variants in cancer predisposition genes other than *PTEN*, and those without germline P/LP variants in the known cancer predisposition genes (Table 1). Importantly, individuals with germline *PTEN* variants had the highest prevalence of cancer compared to the other two groups, including those with germline variants in other cancer predisposition genes (OR, 2.31; 95% CI 1.27–4.14; *P* = 0.007). Additionally, the ages at the first cancer diagnosis were younger in participants with germline *PTEN* variants compared to the other two groups (Table 1). We plotted the cumulative probability of cancer at age of initial diagnosis comparing the three distinct variant groups to show a median age of onset at 48 years (range 8–67 years) for *PTEN* variant carriers, 59 years (range 11–99 years) for carriers of other P/LP cancer-related gene variants, and 61 years (range 3–103 years) for non-carriers (Fig. 2).

### Phenotype Enrichment Analysis (dup: abstract ?)

**Figure 3.**
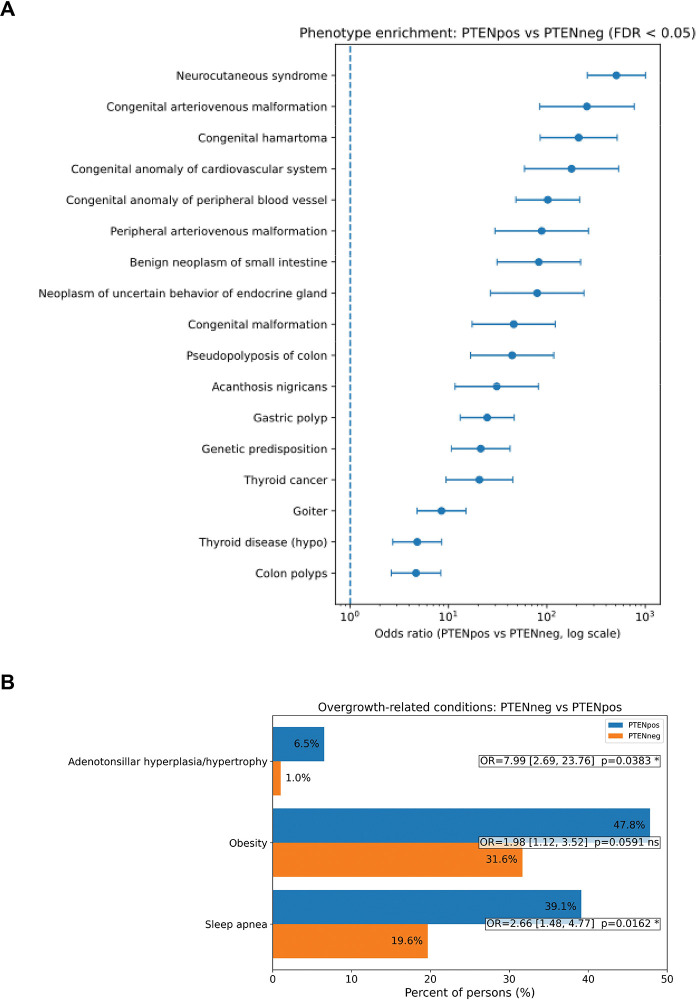
Phenotype Enrichment in Germline *PTEN* Variant Carriers Panel A, Phenotype enrichment in participants with germline *PTEN* variants versus those who are wildtype for *PTEN*. Panel B, Enrichment focused on overgrowth-related conditions.

Intrigued by this observation, we then sought to perform phenotype enrichment analysis between participants with germline P/LP *PTEN* variants versus those who are wildtype for *PTEN*, including those who have germline variants in the 84 other cancer susceptibility genes. We identified known associations with the PHTS phenotype, including neurocutaneous syndrome, congenital anomalies of peripheral blood vessels, congenital hamartoma, goiter, gastrointestinal polyps, endocrine disorders, benign neoplasms, and others (Fig. 3A). Importantly, these phenotypes centered around the overgrowth features of the syndrome. Other previously unrecognized associations include acanthosis nigricans. In an independent targeted analysis, we focused on other overgrowth phenotypes, including hyperplasia of the adenoids and/or tonsils, sleep apnea, and obesity. We identified significant enrichment in adenotonsillar hyperplasia/hypertrophy and sleep apnea in participants with germline *PTEN* variants compared to those without germline *PTEN* variants (Fig. 3B). While obesity was not statistically significant, we observed that extreme obesity with alveolar hypoventilation was significantly enriched in participants with germline *PTEN* variants compared to those without (OR, 19.69; 95% CI 6.07–49.92; *P* < 0.0001).

### Participants with PTEN Variants of Uncertain Significance

Because variants of uncertain significance (VUS) pose a challenge clinically and especially for patient management, we next sought to characterize key features in participants from AoU with *PTEN* VUS. We identified 342 individuals with germline *PTEN* VUS (**Supplementary Table 1**). Notably, compared to participants with P/LP germline *PTEN* variants, carriers of *PTEN* VUS had a lower prevalence of cancer diagnoses (OR, 0.27; 95% CI 0.14–0.53; *P* < 0.001), and an older age at first cancer diagnosis (61 years versus 48 years, *P* < 0.001) (**Supplementary Table 1** and **Supplementary Fig. 1A**). Similarly to carriers of P/LP germline *PTEN* variants, we observe an overrepresentation of broad phenotypes associated with the musculoskeletal system and connective tissues, in addition to endocrine, digestive, genitourinary, and other phenotypes (**Supplementary Fig. 1B**). However, phenotype enrichment analysis of carriers of germline P/LP *PTEN* variants versus those with VUS revealed an overrepresentation of goiter in the former group (OR, 5.8; 95% CI 2.9–11.4; *P* < 0.001).

### Comparison with Cleveland Clinic Patient Series

**Figure 4.**
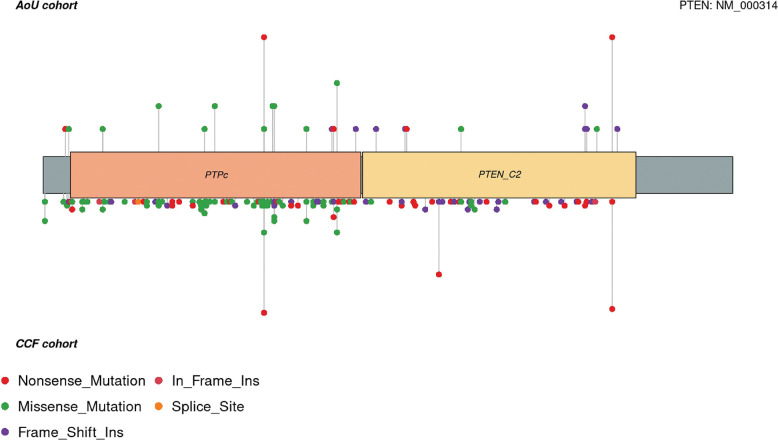
Lollipop Plot of Germline *PTEN* Variants Lollipop heights were normalized in order not to reveal participant count per variant. Plots do not include structural variants. PTPc, protein tyrosine phosphatase catalytic domain; PTEN_C2, C2 domain of the tumor suppressor protein PTEN.

As related to the *PTEN* genotype, we next sought to investigate the *PTEN* genotype spectrum in the 55 participants with 36 unique single nucleotide variants (SNV) and indels (**Supplementary Table 2**). The most predominant variant types were missense variants (46%), followed by nonsense variants (32%), and frameshift deletions or insertions (22%). We then compared these results to the variant spectrum of 487 individuals with germline P/LP *PTEN* variants from Cleveland Clinic PTEN Multidisciplinary Clinic (Fig. 4). In the latter series, the most predominant variant types were missense variants (42%), followed by nonsense variants (27%), and frameshift deletions or insertions (27%). Both series of participants showed an enrichment of hotspot germline *PTEN* variants c.388C > T, p.R130X and c.1003C > T, R335X.

Finally, we focused on cancer phenotypes, which are well-characterized in both participant series from AoU and the PTEN Multidisciplinary Clinic. This analysis included participants with P/LP germline *PTEN* variants, including SNV, indels, and structural variants. In the AoU dataset, 20 of the 46 (43.5%) participants with available EHR data had at least one cancer diagnosis. The most predominant cancers were those of the breast (40%), followed by thyroid (35%), endometrial (20%), skin basal cell carcinoma (15%), and other unspecified cancers (15%). Of those with a cancer diagnosis, 13 (65%) of the participants had SMN. In the Cleveland Clinic series, 215 of the 514 (41.8%) participants had at least one cancer diagnosis. Breast (20%), thyroid (14%), endometrial (7%), kidney (6%), and non-melanoma skin (4%) cancers were the most overrepresented cancers. In this series, 98 (45.6%) of participants with cancer had SMN.

## Discussion

In this study, we leveraged the scale and diversity of the AoU research program to characterize the genotype and phenotype spectrum of PHTS in an unselected population-based setting and compared these findings with individuals from a specialized clinical cohort. Our results demonstrate that individuals with PHTS may present differently in a population-based setting, and that many carriers of germline P/LP *PTEN* variants remain undiagnosed, underscoring the underestimated burden of disease in the general population. For example, while macrocephaly (head circumference greater than the 97th percentile for age) is an important and highly prevalent component manifestation of PHTS,^16, 17^ it was not reported in any of the AoU research participants with germline *PTEN* variants. Importantly, until recently, the true prevalence of PHTS was unknown and was based on a study conducted in the Netherlands that estimated a 1:200,000 prevalence of CS in the Dutch population.^14^ Here, we identify a prevalence of 55/414830 or ~ 1/7500 individuals, which is about 26 folds higher and consistent with estimates from a study focusing on thyroid cancer in PHTS and other thyroid cancer-associated syndromes.^13^

Our comparison of AoU participants with germline *PTEN* variants to individuals with germline variants in the other cancer predisposition genes revealed an elevated cancer prevalence associated with PTEN dysfunction. Importantly, we also observed that carriers of P/LP *PTEN* variants developed cancer at significantly younger ages than both non-carriers and carriers of other cancer predisposition variants. Given that inclusion criteria are identical for all AoU participants, this reinforces prior observations of elevated lifetime cancer risks in PHTS and highlights the importance for earlier, gene-informed surveillance strategies in this population.^3, 6, 7^ Notably, while AoU participants with *PTEN* variants also exhibited enrichment for phenotypes classically associated with PHTS, including endocrine disorders, vascular anomalies, and gastrointestinal polyps, our enrichment analysis revealed under-recognized associations such as vitamin B deficiency, fibromyalgia, and seizure disorders, warranting further investigation into their potential links with PTEN dysfunction.

Comparison of *PTEN* variant spectra across AoU and the Cleveland Clinic PTEN Multidisciplinary Clinic series revealed similar distributions of missense, nonsense, and frameshift variants, including recurrent hotspot variants such as p.R130X and p.R335X. This convergence suggests that while the molecular spectrum of *PTEN* pathogenic variants is consistent across settings, ascertainment biases shape the clinical profiles of identified carriers. Specifically, AoU participants represent a broader and more heterogeneous population, capturing phenotypes less commonly observed in referral-based cohorts. These findings support the utility of large-scale, population-based sequencing efforts to expand our understanding of the full phenotypic spectrum of PHTS beyond cancer- and NDD-enriched settings.

Our study has important clinical and research implications. First, the identification of undiagnosed carriers within AoU validates that PHTS is indeed underdiagnosed and its prevalence underestimated.^13^ Second, the observation of non-component phenotypes through unbiased enrichment analyses raises the possibility that germline *PTEN* variants contribute to a wider range of systemic manifestations than currently recognized, suggesting opportunities for validation studies to delineate these associations. Finally, our data highlight the need for optimizing the identification of PHTS in community settings, particularly in individuals unaffected by cancer and/or NDD.

This study had limitations. Phenotypic manifestations were not uniformly reported between patients from the *PTEN* Multidisciplinary Clinic and Center of Excellence at the Cleveland Clinic and participants from the AoU research program. This precluded us from performing in-depth comparisons and phenotype enrichment analysis beyond well-characterized cancer phenotypes. Relatedly, the AoU cohort lacked detailed information about classical PHTS-associated phenotypes, such as NDD. Conversely, while the CCF series included pediatric and adult patients, including those with NDD across the lifespan, it lacked granular information regarding non-traditional manifestations, such as obesity.

Overall, our study demonstrates the value of population-scale genomic and phenotypic resources for uncovering the true prevalence and breadth of relatively rare syndromes, such as *PTEN*-related disorders. By studying PHTS within an unselected population, we show that its clinical manifestations extend beyond those captured in specialized clinical cohorts and reaffirm its role as a high-penetrance cancer predisposition syndrome with earlier age of onset compared to other heritable cancer syndromes. Future work integrating longitudinal phenotypic data, and exploration of gene–environment interactions will be essential to refining precision surveillance and management strategies for individuals with PHTS.

## Methods

### Research Participants from All of Us Research Program

We analyzed the controlled tier Curated Data Repository (CDR) version 8 dataset of the All of Us (AoU) research program to investigate the phenotypes and genotypes of individuals with *PTEN*-related disorders. By design, the AoU dataset is a diverse, unselected and large-scale dataset that could be representative of the US population across dimensions such as race, ethnic background, sex, geographic region, socioeconomic background, and health status.^18^ As of the cut-off date of October 1, 2023, the program has enrolled 633,547 participants, out of which 353,834 have phenotypic information and 414,830 have short-read whole genome sequencing (srWGS) data. For the purposes of our analysis, we used the ClinVar callset in the hail MatrixTable format with multiallelic sites split into separate records. The AoU ClinVar callset includes variants reported in the ClinVar database, not limited to pathogenic and/or likely pathogenic variants (P/LP), with a total of 2,180,727 single nucleotide variants (SNVs) and indels, with multiallelic sites split into separate records.

The AOU database contains multiple transcripts for each gene. For our analysis, we only used transcripts that were either classified as Matched Annotation from NCBI and EMBL-EBI (MANE) or MANE Plus Clinical.^19^ The following criteria was used to identify P/LP variants. First, we removed any variant whose consequences is annotated as ‘downstream_gene_variant’ or ‘upstream_gene_variant’. Next, any variant labelled as P/LP but also included other ambiguous annotations such as risk factor, uncertain significance or likely allele were manually verified in the ClinVar website.

We identified participants with PHTS associated conditions as those with the following reported conditions in their EHR data: *PTEN* hamartoma tumor syndrome, Cowden syndrome, Lhermitte-Duclos disease, Bannayan syndrome, Proteus syndrome and Proteus like syndrome.

### Phenotype Data Extraction from AoU

The AoU phenotypic data includes different data types collected from participants including data directly collected by the program (e.g., demographics, surveys and physical measurements), data shared through external electronic health records (EHRs), data from wearable devices, and data from biospecimens. The AoU research program uses the Observational Medical Outcomes Partnership (OMOP) Common Data Model (CDM) to store and standardize participants information provided via surveys, physical measurements and EHRs. All data collected are expressed as “concepts” in OMOP. The EHR data includes reported conditions in several EHR codes including SNOMED, ICD10CM, ICD9CM, Nebraska Lexicon, CIEL, and others. To identify relevant phenotypes, we mapped the documented conditions to ICD10CM. Each condition was assigned to one of 22 ICD-10 chapters, including “*Infectious Diseases,” “Neoplasms (Tumors/Cancers),” “Blood & Immune System,” “Endocrine, Nutritional, Metabolic,” “Mental & Behavioral Health,” “Nervous System,” “Eye and Adnexa,” “Ear and Mastoid Process,” “Cardiovascular System,” “Respiratory System,” “Digestive System,” “Skin and Subcutaneous Tissue,” “Musculoskeletal System & Connective Tissue,” “Genitourinary System,”* and *“Congenital Malformations.”* We excluded conditions categorized under the chapters *“Pregnancy, Childbirth, Puerperium,” “Symptoms & Abnormal Clinical Findings,” “Injury & Poisoning,” “External Causes of Morbidity,”* and *“Health Services / Social Circumstances.”* These grouped conditions formed the basis for subsequent phenotype prevalence analyses except when otherwise stated.

### Phenotype Enrichment Analysis

We utilized the standard concept names (grouped manually into related conditions) associated with each phenotype as recorded in the AoU database. We used Fisher’s exact test to calculate odds ratios, 95% confidence intervals, and p-values. To account for multiple hypothesis testing, p-values were adjusted using the false discovery rate (FDR) method. Phenotypes enriched in the *PTEN* cohort were filtered to those with significant p-values (*P* < 0.05). Filtration of prioritized phenotypes shown in Fig. 2 included the following criteria: (1) Requiring at least two *PTEN* variant positive (*PTEN*pos) individuals with the phenotype so ORs are not driven by singletons; (2) Requiring at least 50 individuals (*PTEN*pos plus *PTEN* variant negative [*PTEN*neg]) with the phenotype, so the result is not dominated by rare phenotypes; (3) Requiring a minimum of 10 *PTEN*neg individuals with the phenotype to avoid inflated or infinite ORs and finally (4) Keeping only phenotypes with a maximum 95% CI span (CI_hi/CI_lo) of 25.

### Research Participants from the Cleveland Clinic

Research participants from the Cleveland Clinic were prospectively accrued from September 1, 2005, through January 6, 2022, as part of a prospective follow-up study approved by the Cleveland Clinic institutional review board (IRB protocol 8458).^7^ The study was conducted in agreement with the principles of the Declaration of Helsinki. All participants provided informed written consent to participate. Participants were evaluated at the *PTEN* Multidisciplinary Clinic and Center of Excellence at the Cleveland Clinic (Cleveland, Ohio, USA). This study included both pediatric and adult patients with PHTS accrued from community and academic medical centers throughout North and South America, Europe, Australia, and Asia. Reported cancer diagnoses were documented through pathology reports or verified cancer genetics visits. Baseline information including any cancer history was recorded at the time of consent. Between July 2021 and July 2022, we obtained updated phenotypic information in those who had not routinely seen us in genetics clinic within 3 years.^7, 20^ We reviewed cancer-related health records of patients with PHTS internal to the Cleveland Clinic health system. For the purposes of this study, we prioritized individuals with confirmed P/LP germline *PTEN* variants. *PTEN* variant classifications were ascertained by clinical genetic testing reports where available, ClinVar database classifications, and/or the ClinGen gene-specific criteria for *PTEN* variant curation.^21^

### Statistical Analysis

All statistical analyses were conducted using R studio (version 2024.04.0 Build 735) and Python (3.10.16) programming within the AoU Research Workbench. We analyzed the demographic breakdown of our study cohorts, looking at sex, race, cancer diagnosis status (yes/no), and age at first cancer diagnosis. We utilized the Kruskal-Wallace test to compare age at first cancer diagnosis across cohorts and Mann-Whitney U test for pairwise analyses of age at first diagnosis. We implemented the Chi-Squared test to compare sex, race, and cancer diagnosis breakdowns. A significance threshold of *P* < 0.05 was used throughout the study.

## Supplementary Material

Supplementary Files

This is a list of supplementary files associated with this preprint. Click to download.

• SupplementaryTable2.xlsx

• SupplementaryMaterial.docx

## Figures and Tables

**Table 1:** Comparison of Demographic and Cancer-Related Characteristics by Variant Carrier Status

Variable	Carriers of P/LP *PTEN* Variant N = 55^1^	Carriers of Other 84 P/LP Cancer Gene Variants N = 20,915	Non-Carriers of 85 P/LP Cancer Gene Variants N = 393,860	p-value^3^
**Sex** ^2^				< 0.001
Female	31 (59.6%)	12,736 (61.5%)	237,304 (60.9%)	
Male	21 (40.4%)	7,973 (38.5%)	152,380 (39.1%)	
**Race**				< 0.001
American Indian or Alaska Native	0 (0%)	239 (1.1%)	5,515 (1.4%)	
Asian	*	435 (2.1%)	13,051 (3.3%)	
Black or African American	*	2,543 (12%)	69,989 (17.8%)	
Middle Eastern or North African	0 (0%)	95 (0.5%)	2,256 (0.6%)	
More than one population	*	896 (4.3%)	16,920 (4.3%)	
Native Hawaiian or Other Pacific Islander	0 (0%)	21 (0.1%)	435 (0.1%)	
Other/Unspecified	*	3,911 (19%)	74,508 (18.9%)	
White	36 (65%)	12,775 (61%)	211,186 (53.6%)	
**Cancer Diagnosis** ^4^				< 0.001
No	26 (56.7%)	11,058 (75.0%)	220,688 (81.3%)	
Yes	20 (43.5%)	3,688 (25.0%)	50,882 (18.7%)	
**Age at First Cancer Diagnosis** ^5^	46 (8–67)	59 (9–99)	61 (3–105)	< 0.001

1 Includes 2 individuals with structural variant.

2 We only report sex count for male and female.

3 p-values calculated using Kruskal-Wallis test for age and Chi-Squared test for others.

4 Restricted to participants who have both srWGS and EHR data, therefore the sum may not equal N.

5 Refers to first of any kind of cancer; n (%), Median (Min – Max).

## Data Availability

All of Us (AoU) data analyses were performed using the AoU Controlled Tier Dataset v8 dataset and executed using the AoU Research Workbench. All other raw data are available from the corresponding author on reasonable request. All other bioinformatics and statistical tools are publicly available and are described above.

## References

[1] Yehia L, Eng C. PTEN Hamartoma Tumor Syndrome. In: GeneReviews((R)) (eds Adam MP, et al.) (1993).

[2] Yehia L, Keel E, Eng C. The Clinical Spectrum of PTEN Mutations. Annu Rev Med 71, 103–116 (2020). 31433956 10.1146/annurev-med-052218-125823

[3] Tan MH, Mester JL, Ngeow J, Rybicki LA, Orloff MS, Eng C. Lifetime cancer risks in individuals with germline PTEN mutations. Clin Cancer Res 18, 400–407 (2012). 22252256 10.1158/1078-0432.CCR-11-2283 PMC3261579

[4] Bubien V, High cumulative risks of cancer in patients with PTEN hamartoma tumour syndrome. J Med Genet 50, 255–263 (2013). 23335809 10.1136/jmedgenet-2012-101339

[5] Nieuwenhuis MH, Cancer risk and genotype-phenotype correlations in PTEN hamartoma tumor syndrome. Fam Cancer 13, 57–63 (2014). 23934601 10.1007/s10689-013-9674-3

[6] Hendricks LAJ, Cancer risks by sex and variant type in PTEN hamartoma tumor syndrome. J Natl Cancer Inst 115, 93–103 (2023). 36171661 10.1093/jnci/djac188

[7] Yehia L, Longitudinal Analysis of Cancer Risk in Children and Adults With Germline PTEN Variants. JAMA Netw Open 6, e239705 (2023). 37093598 10.1001/jamanetworkopen.2023.9705 PMC10126871

[8] Ngeow J, Stanuch K, Mester JL, Barnholtz-Sloan JS, Eng C. Second malignant neoplasms in patients with Cowden syndrome with underlying germline PTEN mutations. J Clin Oncol 32, 1818–1824 (2014). 24778394 10.1200/JCO.2013.53.6656 PMC4039869

[9] Hendricks LAJ, The risk of a second primary cancer in PTEN Hamartoma Tumor Syndrome (PHTS). Genet Med, 101467 (2025). 40433764 10.1016/j.gim.2025.101467

[10] Butler MG, Subset of individuals with autism spectrum disorders and extreme macrocephaly associated with germline PTEN tumour suppressor gene mutations. J Med Genet 42, 318–321 (2005). 15805158 10.1136/jmg.2004.024646 PMC1736032

[11] Satterstrom FK, Large-Scale Exome Sequencing Study Implicates Both Developmental and Functional Changes in the Neurobiology of Autism. Cell 180, 568–584 e523 (2020). 31981491 10.1016/j.cell.2019.12.036 PMC7250485

[12] Trost B, Genomic architecture of autism from comprehensive whole-genome sequence annotation. Cell 185, 4409–4427.e4418 (2022). 36368308 10.1016/j.cell.2022.10.009 PMC10726699

[13] White SL, Population prevalence of the major thyroid cancer-associated syndromes. J Clin Endocrinol Metab, (2025). 10.1210/clinem/dgaf236 PMC12623018 40231587

[14] Nelen MR, Novel PTEN mutations in patients with Cowden disease: absence of clear genotype-phenotype correlations. Eur J Hum Genet 7, 267–273 (1999). 10234502 10.1038/sj.ejhg.5200289

[15] Idumah G, Newell D, Hadrys M, Ribaudo I, Ni Y, Arbesman J. Pathogenic Germline Variants in Cancer Susceptibility Genes. JAMA, (2025). 10.1001/jama.2025.16372 PMC12532028 41100108

[16] Mester JL, Tilot AK, Rybicki LA, Frazier TW, 2nd, Eng C. Analysis of prevalence and degree of macrocephaly in patients with germline PTEN mutations and of brain weight in Pten knock-in murine model. Eur J Hum Genet 19, 763–768 (2011). 21343951 10.1038/ejhg.2011.20 PMC3137495

[17] Drissen M, Schieving JH, Schuurs-Hoeijmakers JHM, Vos JR, Hoogerbrugge N. Red flags for early recognition of adult patients with PTEN Hamartoma Tumour Syndrome. Eur J Med Genet 64, 104364 (2021). 34637944 10.1016/j.ejmg.2021.104364

[18] All of Us Research Program I, The “All of Us” Research Program. N Engl J Med 381, 668–676 (2019). 31412182 10.1056/NEJMsr1809937 PMC8291101

[19] Morales J, A joint NCBI and EMBL-EBI transcript set for clinical genomics and research. Nature 604, 310–315 (2022). 35388217 10.1038/s41586-022-04558-8 PMC9007741

[20] Yehia L, Extended spectrum of cancers in PTEN hamartoma tumor syndrome. NPJ Precis Oncol 9, 61 (2025). 40050354 10.1038/s41698-025-00847-3 PMC11885834

[21] Mester JL, Gene-specific criteria for PTEN variant curation: Recommendations from the ClinGen PTEN Expert Panel. Hum Mutat 39, 1581–1592 (2018). 30311380 10.1002/humu.23636 PMC6329583

